# ﻿Confirmed occurrence of the tribe Apameini (Lepidoptera, Noctuidae, Noctuinae) in the Neotropical region: a new genus endemic to Costa Rican montane forests

**DOI:** 10.3897/zookeys.1114.84361

**Published:** 2022-07-27

**Authors:** B. Christian Schmidt

**Affiliations:** 1 Canadian National Collection of Insects, Arachnids and Nematodes, Agriculture and Agri-Food Canada, K.W. Neatby Bldg., 960 Carling Ave., Ottawa, ON, K1A 0C6, Canada Canadian National Collection of Insects Ottawa Canada

**Keywords:** Borer, *Chusquea*, Cloud forest, Monteverde, Talamancan montane forest

## Abstract

The genus *Nublapamea***gen. nov.** is described (type species: *Tracheaaltivolans* Schaus), here determined to belong to the primarily temperate Holarctic tribe Apameini (Noctuidae: Noctuinae). Currently known only from mid- to upper elevation montane forests of Costa Rica, *Nublapamea* is a disjunct southern extension of a largely northern hemisphere temperate region fauna. The life history of *Nublapameaaltivolans* is unknown; it may be associated with chusquea bamboo (*Chusquea* Kunth), as most Apameini are dietary specialists on graminoids.

## ﻿Introduction

The tribe Apameini (Noctuidae: Noctuinae) currently includes 31 named genera in North America, out of a global total of at least 70 genera (Zilli et al. 2005, 2009; Lafontaine and Schmidt 2010). The majority of apameines are found in the temperate regions of the Northern Hemisphere, with the greatest diversity in temperate-subtropical Asia, although endemic genera occur also in Africa (including Madagascar) and the Indo-Australian Region (Zilli et al. 2009). No apameines are known from the Neotropical Region (Zilli et al. 2005), although true *Apamea* Ochsenheimer reach central Mexico (Mikkola et al. 2009). The few South American species historically placed in temperate-Holarctic apameine genera likely or certainly require generic and tribal re-assignment (Poole 1989).

The biology of apameines is unique within Noctuidae; they are well-known for the specialized endophagous feeding mode of many genera upon plant stems, roots, and rhizomes, especially of graminoid plants and other monocots. Several (possibly independent) apameine lineages have switched to non-graminoid host plants, most notably the *Papaipema* group of genera that includes both Old- and New World representatives such as *Hydraecia* and *Amphipoea*. Morphologically, apameines are immediately recognizable by the unique structure of the highly modified female ovipositor, among other traits. The dietary specialization and high habitat fidelity of apameines combine to make this a relatively under-studied group; the taxonomic knowledge gap among apameines is disproportionately large for the otherwise well-known eastern North American noctuid fauna (e.g., Quinter and Sullivan 2014).

Contrary to latitudinal diversity gradients in many insect groups, the Noctuidae do not increase in diversity in the Neotropics; in fact, there are about 35% fewer recognized species in the Neotropical versus the Nearctic realm (Poole 1989; Lafontaine and Schmidt 2010). Despite the limited diversity of Neotropical Noctuidae, their taxonomy and systematic composition remains poorly known, essentially unchanged from the time of Hampson’s (1898–1913) artificial classification (for a review of historic changes to noctuid classification, see Mitchell et al. (2006) and references therein). Exceptions include recent molecular studies that have incorporated a limited number of Neotropical genera, nonetheless making significant inroads to clarifying the major Noctuidae lineages present in the Neotropics, such as the discovery of the basal subfamily Dyopsinae and its bizarre constituent genera (Zahiri et al. 2013; Keegan et al. 2021), the equally odd *Vespola* Walker group of genera related to Bagisarinae (Zahiri et al. 2013), and the circumscription of entirely new subfamilies such as Cobubathinae and Cropiinae (Keegan et al. 2021). As part of ongoing work to revise the genera of Nearctic Apameini and placing Neotropical genera into a systematic framework, examination of the genus *Trachea* Ochsenheimer revealed that few, if any, of the Neotropical species truly belong to this Holarctic genus, and led to the surprising find that “*Trachea*” *altivolans* belongs to the Apameini. *Trachea* is currently placed in the tribe Dypterygiini (Lafontaine and Schmidt 2010; Keegan et al. 2021). A new genus is herein described to accommodate “*Trachea*” *altivolans* and to facilitate future study of the taxonomy and biogeography of the New World Apameini.

## ﻿Methods and materials

Specimens examined include those deposited in the Canadian National Collection of Insects, Arachnids and Nematodes (CNC), Ottawa, Canada, and DNA barcodes and associated voucher photos are available at The Barcode of Life Data System (Ratnasingham and Hebert 2007). Genitalia were prepared following the methods of Lafontaine (2004) and Jaeger (2017). Cleaned, stained genitalia were stored and examined in 30% ethanol, and slide-mounted in Euparal before being photographed using a Leica DFC450 digital camera.

## ﻿Systematics

### ﻿ Nublapamea gen. nov.

Taxon classificationAnimaliaLepidopteraNoctuidae

68D1C901-0D30-5B85-AEBA-711EC8321F5F

http://zoobank.org/66607E89-8801-4B30-9FE3-63056D3879A0

**Figure 1.**
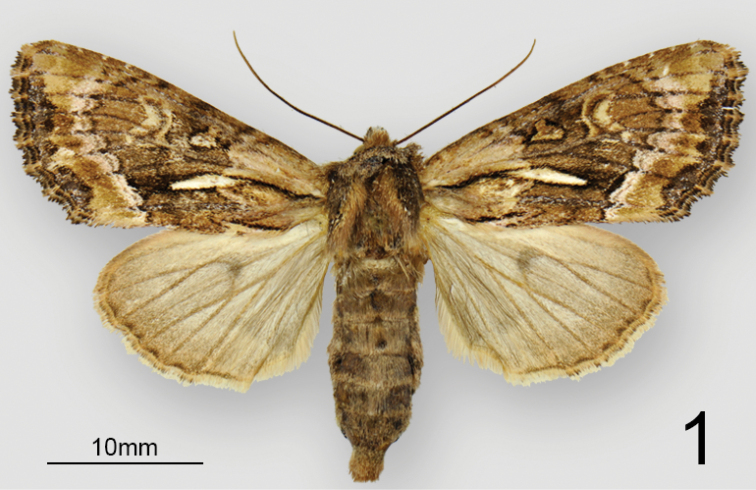
Adult habitus of female *Nublapameaaltivolans*.

**Figure 2.**
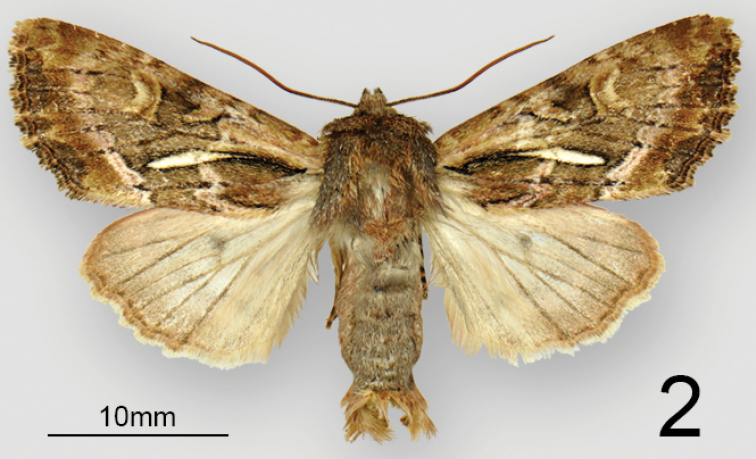
Adult habitus of male *Nublapameaaltivolans*.

**Figure 3.**
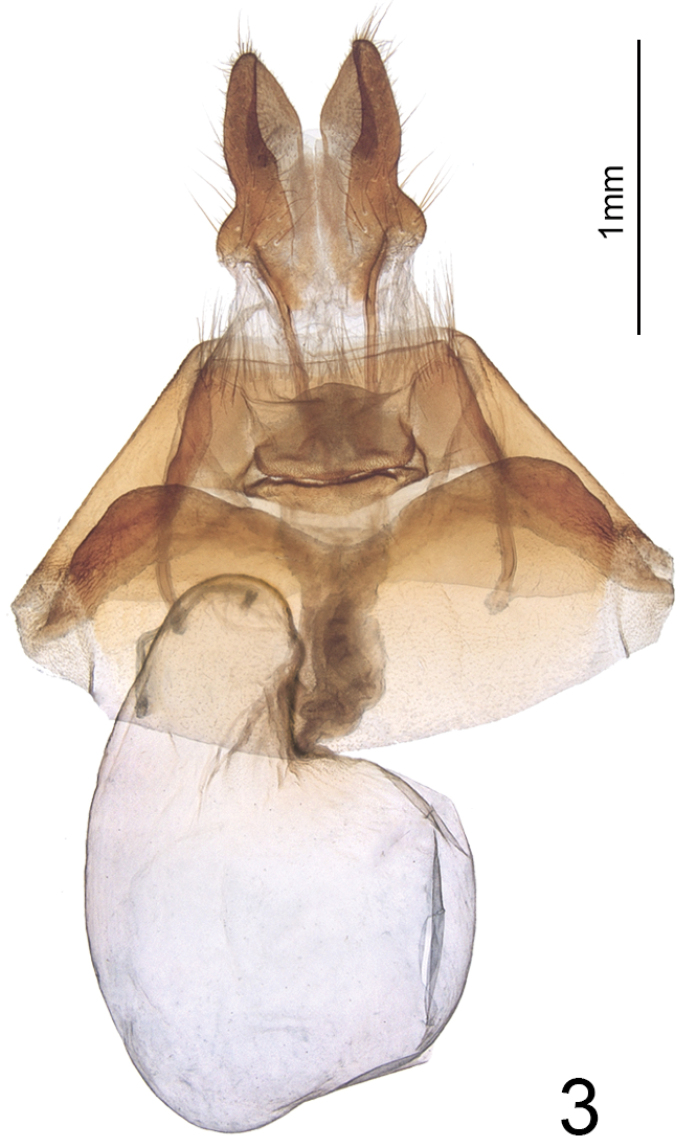
Female genitalia of *Nublapameaaltivolans*.

**Figure 4.**
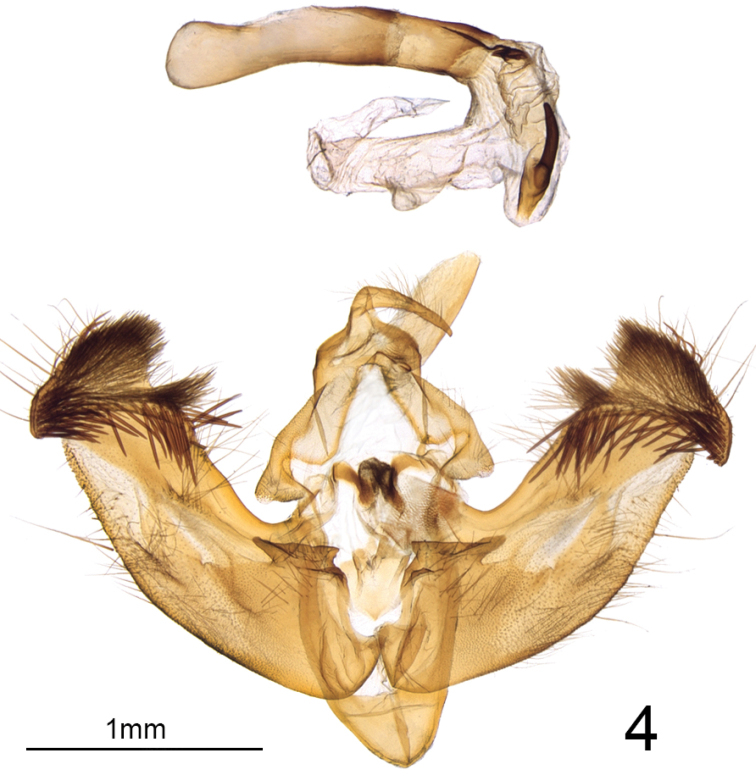
Male genitalia of *Nublapameaaltivolans*.

**Figure 5.**
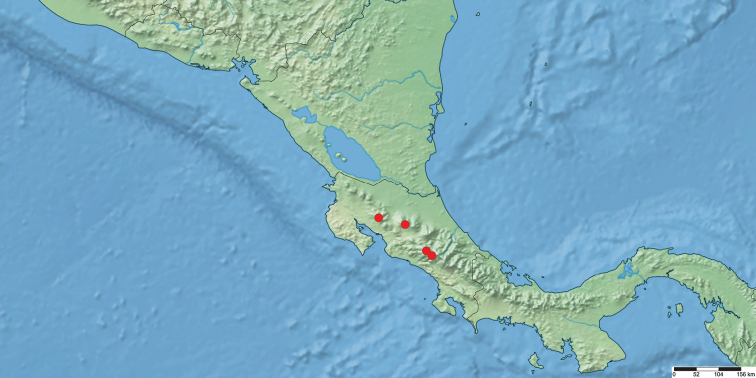
Distribution of examined specimens of *Nublapameaaltivolans*.

Figs 1 , 2 , 3 , 4 , 5

#### Type species.

*Tracheaaltivolans* Schaus, 1911: 96; TL: Volcano Poas [Alajuela Prov., Costa Rica]. USNM [examined].

#### Included species.

*Tracheaaltivolans* Schaus.

#### Diagnosis.

Most of the autapomorphies of the tribe Apameini are based on adult genitalic morphology and were reviewed by Fibiger and Lafontaine (2005), with an updated tribal concept modified slightly through the removal of the Arzamini as a separate tribe (Lafontaine and Fibiger 2006). *Nublapamea* exhibits four key autapomorphies of the Apameini: ovipositor heavily sclerotized and dorsoventrally flattened, with a unique profile resembling rabbit ears when viewed ventrally; two well-sclerotized elongate platelets (sometimes termed “rods”) situated ventrally in the integument between the papillae anales; pleural sclerite of male genitalia comprising a twisted, helical ribbon (the “double helix” of Fibiger and Lafontaine (2005), although technically not double).

The morphology of *Nublapamea* male genitalia is unlike any other Apameini genera in that the clasper, digitus and ampulla are seemingly absent (presumably a result of the extreme reduction of these structures), combined with a very robust valve with only a slight narrowing of the neck of the cucullus. A heavily spinose ridge extends obliquely across the inner surface of the valve from the caudoventral apex of the cucullus to the dorsal edge of the costa, similar to some species of *Apamea* (*A.verbascoides* (Guenée), *A.inebriata* Ferguson) although with more diminutive spines. A reduction of clasper and digitus occurs also in *Resapamea*, but the two genera otherwise differ in most other genitalic traits and do not associate in DNA barcode sequence data. *Nublapameaaltivolans* is among the largest New World Apameini, with a forewing length of up to 25 mm. The forewing color and pattern most closely approaches some western North American *Apamea*, such as *A.antennata*, *A.centralis* and *A.siskiyou*; however, the large, ivory-filled claviform stigma easily distinguishes *Nublapamea* from any *Apamea*. The similarity to *Apamea* is superficial only, as *Nublapamea* differs dramatically from all known species of *Apamea* in genitalic structure and lacks the autapomorphies of that genus (Mikkola et al. 2009; Zilli et al. 2009).

#### Description.

***Head*** – Male and female antennae simple, setose-ciliate, ~68 segments, flattened ventrally and convex dorsally (D-shaped in cross-section). Eye smooth, round. Labial palpus, upcurved, first segment 0.7 × length of second segment; third segment 0.5 × length of second and directed more anteriorly. Frons evenly convex, unmodified; haustellum well developed. ***Thorax*** – Mesoscutellar crest scarcely differentiated, metascutellar tuft absent. ***Forewing*** (Figs 1, 2) – shape and pattern elements typically noctuine, most similar to *Apameaantennata* group; venation as illustrated in Mikkola et al. (2009) except that (R5+(R3+R4)) arises from a common stalk that is 1/3 the length of the areole, with the stalk of (R3+R4) about 1/2 the length of the areole; forewing length up to 25 mm. ***Hindwing*** – vein M2 somewhat reduced but clearly visible, originating from the bottom third of the cell. Legs – spination and proportions typically apameine: tibia lacking spines, tibial spur formula 0-2-4, epiphysis 0.6 × length of tibia; tarsus with three rows of spiniform setae on first two proximal tarsomeres; four irregular rows on distal three tarsomeres. ***Abdomen*** – Pronounced dorsal setal tufts on A2 and A3, smaller tufts on A4 and A5; male basal abdominal brushes absent. ***Female genitalia*** (Fig. 3) – Papillae anales dorsoventrally flattened, apical 2/3 rounded-triangular, base defined by a pronounced sub-basal constriction; surface moderately setose and densely microspinulose. Two elongate sclerotized plates present between papillae anales. Posterior and anterior apophyses 1.1 × and 0.7 × length of papillae anales, respectively, with slightly spatulate apices. Lamella antevaginalis well sclerotized, with a broad, round medial concavity. Ostium an irregular transverse slit, heavily microspinulose. Ductus bursae 3 × as long as wide, heavily rugose, appearing thick or slightly more sclerotized than corpus; ductus bursae joined at junction of corpus and appendix bursae on right side. Corpus bursae membranous, globose-pyriform, 2 × as long as wide, signa absent. Appendix bursae unmodified and arising posteriorly on left, approximately 1/3 size of corpus bursae. ***Male genitalia*** (Fig. 4) – Uncus of moderate length, approximately 7 × longer than medial width, laterally compressed and evenly tapering to a downcurved, blunt apex; sparsely covered with fine, long setae. Tegumen forming an offset, broad base at uncus, with rounded-triangular peniculum laterally; vinculum a rounded “V,” saccus approximately as long as wide. Juxta a trapezoidal shield, length equal to width; anellar arms not fused. Valve robust, ~3 × longer than wide, tapering only very slightly towards base of cucullus and therefore lacking a distinct subapical “neck;” ventral margin of valve evenly convex; dorsal margin essentially straight beyond base. Cucullus elongate-triangular and broad-based, corona consisting of 4–6 curved spiniform setae; inner surface of cucullus densely covered in long, straight bristle-like setae; prominent ridge extending from caudoventral point of cucullus obliquely across the inner surface of the valve to the dorsal edge of the costa, beset with > 50 long, straight spine-like setae which are directed basad. Sacculus with basal saccular process (not the clavus; Crabo et al. 2013) consisting of an angular, anvil-shaped lobe, with an additional ridge-like prominence on base of lobe; clasper scarcely discernible, a minute rounded ridge; ampulla of clasper a minute nodule bearing 5–6 setae; editum scarcely discernible as a slightly raised bump bearing 20–25 long, thin bristle-like setae; digitus absent. Aedeagus 5 × longer than wide, with slight ventrad curvature, with thin sclerotized band extending onto base of vesica and adjoining sclerotized plate of 6–7 stout, ventrally-projecting spines that are closely set and sub-parallel; vesica slightly rugose and sclerotized at base, and with a sub-basal and medial diverticulum; sub-basal diverticulum with a large tine-like spine that is directed basad; vesica length 0.8 × that of aedeagus.

#### Etymology.

*Nublapamea* is a composition derived from *bosque nublado*, the Latin American term for the cloud forest habitat of the type species, and *Apamea*, the type genus of the tribe Apameini.

#### Remarks.

Cursory examination of other Neotropical species with externally similar facies, particularly those currently placed in *Trachea*, *Paratrachea*, and *Heterochroma* did not reveal other potential congeners. *Nublapameaaltivolans* in the BOLD record database (as *Tracheaactivolans*; *sic*) are assigned to BIN number BOLD:AAE8386 (http://v4.boldsystems.org).

#### Biology and distribution.

The immature stages, larval biology, and host plants of *Nublapameaaltivolans*, the sole species currently in the genus, are unknown. The ecology of most Apameini is closely linked to graminoid monocots, with the characteristic female ovipositor modified to insert eggs into various parts of the host plant, including between the leaf blade and stalk, and within seed heads. Since many apameine species are closely linked to specific graminoid hosts, it may be that *Nublapamea* is associated with *Chusquea* (Poaceae: subfamily Bambusoideae), a common graminoid of Neotropical cloud forests. Although no other apameines are known to utilize *Chusquea*, several North American genera are dietary specialists on *Arundinaria*, also a bambusoid grass (subfamily Bambusoideae). The distribution is limited to the Talamancan montane forest ecoregion of montane Costa Rica, between elevations of 1500 and 3300 m (Fig. 5).

## ﻿Discussion

Neither DNA barcode data nor morphology provide many clues to possible relationships of *Nublapamea* to other New World genera. Unlike most of the larger Noctuinae tribes, apameines have an unusually high proportion of small genera (three species or fewer; Zilli et al. 2005), a symptom that appears to truly reflect evolutionary patterns (as opposed to inadequate taxonomy). Exceptions include the large genera *Apamea* and *Papaipema*, but *Nublapamea* is not closely related to either. Comparison of *Nublapameaaltivolans* barcodes to those of all other Nearctic Noctuidae (Zahiri et al. 2017) consistently places *Nublapamea* among other Apameini genera, albeit with a highly variable topology, with nearest-neighbour distances of at least 5.2%. None of the Nearctic genera exhibit clear morphological traits that would indicate a relationship to *Nublapamea*. Resolution of evolutionary relationships among New World and indeed global Apameini must await other molecular techniques.

High elevation habitats of central America, including cloud forests, are well known for harboring southern extensions of north-temperate plant genera, including *Alnus*, *Myrica*, *Juglans*, and *Quercus* (Graham 2010). Knowledge of the Lepidoptera fauna of this biogeographically important region remains fragmentary, but similar distribution patterns are known in *Acleris* (Tortricidae; Brown and Nishida 2008), *Euxoa* (Noctuidae; Lafontaine 1987) and *Acronicta* (Noctuidae; Schmidt and Anweiler 2020); *Nublapamea* represents the only member of the tribe Apameini currently known to occur in the Neotropics, although many questions remain unanswered: what are the life history and host plant? Is *Nublapamea* truly monotypic? What is the full geographic extent of the genus, and how is it related to other New World genera? These questions must await further study of the Apameini, and of the cloud forest fauna of meso-America.

## Supplementary Material

XML Treatment for Nublapamea
